# Multi-Feature Facial Complexion Classification Algorithms Based on CNN

**DOI:** 10.3390/biomimetics10060402

**Published:** 2025-06-13

**Authors:** Xiyuan Cao, Delong Zhang, Chunyang Jin, Zhidong Zhang, Chenyang Xue

**Affiliations:** State Key Laboratory of Extreme Environment Optoelectronic Dynamic Testing Technology and Instrument, North University of China, Taiyuan 030051, China; caoxiyuan@nuc.edu.cn (X.C.); s202206076@st.nuc.edu.cn (D.Z.); sz202306012@st.nuc.edu.cn (C.J.)

**Keywords:** facial complexion classification, CNN, machine learning, multi-feature

## Abstract

Variations in facial complexion serve as a telltale sign of underlying health conditions. Precisely categorizing facial complexions poses a significant challenge due to the subtle distinctions in facial features. Three multi-feature facial complexion classification algorithms leveraging convolutional neural networks (CNNs) are proposed. They fuse, splice, or independently train the features extracted from distinct facial regions of interest (ROI), respectively. Innovative frameworks of the three algorithms can more effectively exploit facial features, improving the utilization rate of feature information and classification performance. We trained and validated the three algorithms on the dataset consisting of 721 facial images that we had collected and preprocessed. The comprehensive evaluation reveals that multi-feature fusion and splicing classification algorithms achieve accuracies of 95.98% and 93.76%, respectively. The optimal approach combining multi-feature CNN with machine learning algorithms attains a remarkable accuracy of 97.78%. Additionally, these experiments proved that the multidomain combination was crucial, and the arrangement of ROI features, including the nose, forehead, philtrum, and right and left cheek, was the optimal choice for classification. Furthermore, we employed the EfficientNet model for training on the face image as a whole, which achieves a classification accuracy of 89.37%. The difference in accuracy underscores the superiority and efficacy of multi-feature classification algorithms. The employment of multi-feature fusion algorithms in facial complexion classification holds substantial advantages, ushering in fresh research directions in the field of facial complexion classification and deep learning.

## 1. Introduction

Inspection with traditional Chinese medicine (TCM), as a non-invasive diagnostic modality, offers a convenient way to assess health status through a patient’s complexion, tongue coating, and overall vitality. The facial region, which carries holographic information, maps the whole body to reflect the diseases and lesions of different viscera [1]. Facial complexion, as a pivotal symptom in traditional and modern medical assessments, plays a vital role in offering practitioners a wealth of invaluable insights into a patient’s health status. Typically, practitioners who rely on visual inspection to assess facial complexion are limited by their professional accomplishments and are simultaneously affected by volatile external environments [2]. Therefore, the quantitative analysis of facial complexion classification by computer vision technology provides an objective and standardized criterion for TCM inspection [3,4]. The pathological complexion is generally divided into five kinds: cyan, red, sallow, pallid, and dark [5,6]. Each complexion is related to the corresponding organ and the degree of lesion [7]. The subtle inter-individual variations in facial characteristics make complexion classification a clinically challenging task, particularly in TCM diagnostic applications. Therefore, a crucial aspect lies in accurately categorizing the complexion.

Conventional facial complexion classification methods involve the application of machine learning algorithms to train and classify features extracted from different color spaces [8]. Li et al. designed a facial complexion collection and recognition system, which collects data through a camera obscura and circular LED lights [9]. Based on the traditional Chinese medicine (TCM) theory, they also devised a Five-Color Complexion Chart for observing facial complexions. Lin et al. employed KNN, SVM, and ELM for facial complexion recognition by utilizing primary features extracted by PCA (principal component analysis) in different color spaces [10,11,12]. Zheng et al. divided the hue, saturation, and luminance channels into different numbers of bins, then computed the corresponding feature vectors, combined them together as feature vectors, and finally, classified them using multi-classes SVM as a classifier [13]. Wang et al. utilized the SVM algorithm for zygomatic color recognition by determining the percentage of red pixels on the face [14]. Xu et al. first utilized the Gaussian Mixture Model (GMM) to select complexion pixels corresponding to skin tones, thereby eliminating other interfering factors [15]. Subsequently, they employed a Support Vector Machine (SVM) classifier with a linear kernel function to classify human faces. Sun et al. divided face complexion classification into overall classification and local classification by using the Dlib library for segmentation of different facial regions and exploited K-Means or SVM algorithms for face complexion classification, respectively [16]. After manually extracting color features from face complexion images, Li et al. converted them into one-dimensional features and constructed a classification model [17]. Then, they employed the supervised Latent Dirichlet Allocation (sLDA) method for face complexion recognition. Overall, conventional methods rely on feature design. Excellent features can accurately lead to beneficial information, whereas relatively poor features cannot bring about useful training information.

Deep learning has been a hot research direction in the field of machine learning in recent years. Deep learning-based convolutional neural networks are able to avoid the manual feature extraction process that relies on expert experience in traditional machine learning through its hierarchical feature learning mechanism [18,19]. It can automatically mine multi-level and non-linear pathological features from raw facial images that are difficult to be recognized by the human eye [20]. Hou et al. improved the CNN-based CaffeNet model to classify tongue colors, thereby demonstrating the superiority of deep learning methods over conventional approaches in terms of practicality and accuracy [21]. Alami et al. proposed the utilization of pure quaternions to represent the RGB color components of images for face recognition by constructing a deep learning model [22]. Lin et al. employed convolutional neural network models such as AlexNet, VGGnet, and ResNet for facial complexion classification, and subsequently compared their performance with various machine learning methods [23]. All of their methods use the entire facial image as input, resulting in the underutilization of feature information and interference from other factors. Zhao et al. first manually extracted color, texture, and luster features from facial images and concatenated them [24]. Then, MobileViT was utilized for self-learning feature extraction. Finally, the features from both parts were concatenated before classification, ultimately achieving an accuracy rate of 94.12%. This approach of manual feature extraction is subjective, and the use of a deep learning model still involves extracting features from the entire face.

To improve feature utilization and accurately classify face colors, we introduce three multi-feature algorithms for facial complexion classification: a multi-feature fusion classification algorithm, a multi-feature splicing algorithm, and a multi-feature machine learning algorithm. The core contributions and innovative aspects of the research are primarily manifested in the following three aspects:

(1) We leverage the Dlib library to extract facial keypoints and partition them into six distinct regions of interest (ROI), which is analogous to how traditional Chinese medicine conducts a diagnosis by evaluating multiple local facial features. This concept is also reflected in the holographic mapping phenomenon observed in biological systems [25].
(2) Deep learning algorithms are trained by integrating various visual cues, much like how biological neural networks process multimodal sensory inputs [26]. Therefore, we apply convolutional neural networks to extract features for each ROI.
(3) We proposed three facial complexion classification algorithms that have never been developed by others. The multi-feature fusion algorithm integrates diverse features into channels and incorporates grayscale information that reflects the multimodal information integration properties of the biological visual system. The multi-feature splicing algorithm splices the different ROI sections together for training, while the multi-feature machine learning classification algorithm employs a hybrid approach of deep learning and machine learning for classification.

We tested and compared the three algorithms in the same environment. The multi-feature fusion algorithm attained a classification accuracy of 95.98%, the multi-feature splicing algorithm achieved a maximum accuracy of 93.76%, and the machine learning algorithm excelled with an accuracy of 97.78%. Finally, we delved into the correlation and importance of different ROI features. The bio-inspired approach bridges AI-driven diagnostics with traditional medical expertise and improves classification performance.

This paper is organized as follows. In Section 2 we will present our dataset and the specific steps of the proposed method. In Section 3, we will present the experiment results of our methods. Finally, we will make a conclusion in Section 4.

## 2. Method Overview

In this section, we will describe the datasets and proposed methods in detail. The dataset is first segmented into separate face images for full facial complexion analysis and specific ROI segmentation. Secondly, three multi-feature classification algorithms are presented, namely multi-feature fusion classification algorithm, multi-feature splicing classification algorithm, and multi-feature classification algorithms with machine learning.

### 2.1. Dataset

The dataset comprises an extensive collection of facial images, curated to ensure the highest quality and consistency of the captured images. We collaborated with Shanxi University of Traditional Chinese Medicine to procure and assemble these facial datasets. To ensure the objectivity and effectiveness of classification, facial image data were collected using specialized equipment under controlled lighting conditions. The color temperature of the instrument was set above 5500 K, the illuminance was around 3000, and the color rendering index was 90%.

**Figure 1:**
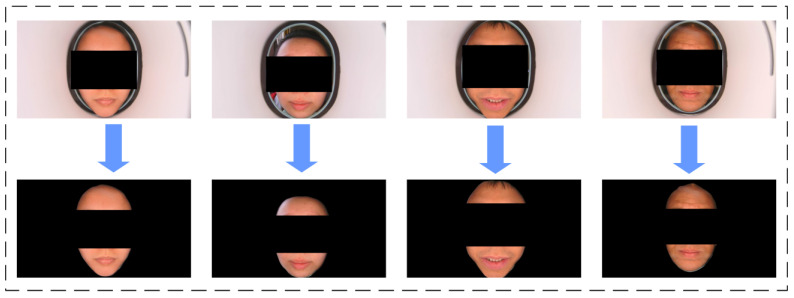
Examples of face before and after cutting. Although we had obtained consent from the patients at the time of photo collection, we still applied mosaic processing to their portraits.

During the collection process, we maintained the shooting environment’s standards and stability, ensuring the pristine quality of facial images. We collected facial images from a diverse group of 600 patients, whose ages ranged from 18 to 70 years old, with each image boasting a resolution of 3840 × 2160 pixels, guaranteeing clarity and detail. The categorization of facial complexions was a collaborative effort undertaken by two experienced traditional Chinese medicine (TCM) practitioners. We discussed and labeled them simultaneously to ensure the authenticity of the classification. Given the challenges in collecting data on cyan facial complexion, we segmented it into five distinct categories: dark, red, pallid, sallow, and normal. Subsequently, as depicted in Figure 1, the gathered facial data underwent preprocessing using the Deeplab3+ model [27], effectively eliminating background information and yielding a segmented facial dataset tailored for experimental purposes. To further enhance the dataset’s diversity and robustness, we augmented the processed data through horizontal flipping. This augmentation process ultimately culminated in a final dataset comprising 721 images, with the ratio of the five categories standing at 131:134:168:160:128. To ensure the reliability and generalizability of our findings, we divided the dataset into training and testing sets at a ratio of 8:2.

### 2.2. Method

We will describe the three proposed methods in detail for facial complexion classification, namely the multi-feature fusion algorithm, the multi-feature splicing algorithm, and the multi-feature classification algorithms combined with machine learning, respectively. They combine six features (nose, forehead, philtrum, chin, right cheek, and left cheek) for classification. According to the theory of TCM, facial complexion is systematically classified into five canonical categories: dark, red, pallid, sallow, and normal.

#### 2.2.1. Multi-Feature Fusion Classification Algorithm

**Figure 2:**
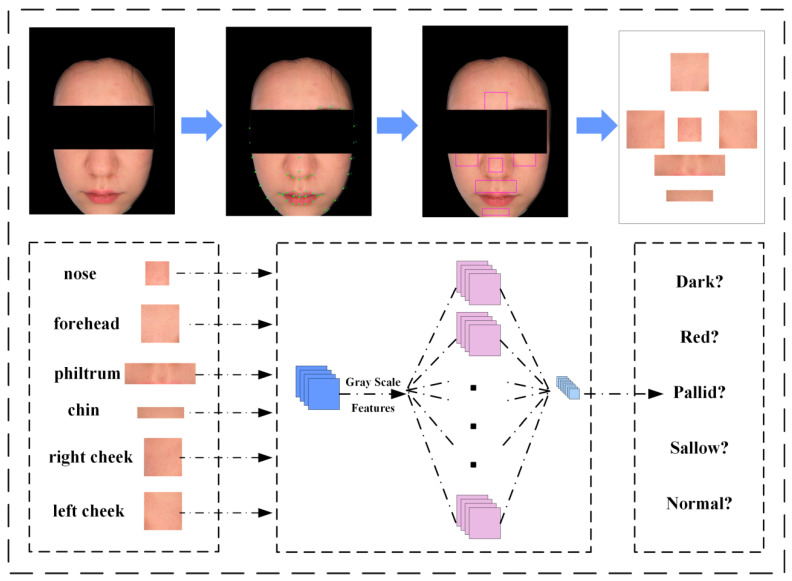
The framework of the multi-feature fusion classification algorithm, where the network used is EfficientNet-B0.

(a) Utilizing the Dlib library, we calibrated 68 crucial facial landmarks. Based on the coordinates of these key points, the face is segmented into six representative ROIs as illustrated in Figure 2: nose, forehead, philtrum, chin, right cheek, and left cheek [28]. We engaged in discussions with practitioners of TCM and ultimately decided to adopt these ROIs. While everyone possesses the same facial landmarks, variations in facial dimensions lead to disparities in the sizes of the six ROIs. Consequently, we determine the coordinates of each ROI by selectively referencing the keypoints within the captured images, ensuring consistency in ROI shapes. For example, the chin area is calculated using the coordinates of keypoints 9th, 56th, 58th, and 60th.

(b) In this phase, we integrated diverse features from the Regions of Interest (ROIs) by leveraging a Convolutional Neural Network (CNN) for feature extraction. In addition to merging the RGB color data of the ROI segments, we incorporated grayscale information to enrich the feature set. The input image was resized to 224 × 224 pixels, with an initial channel count of 4. The primary function of the CNN at this stage is to individually extract features from the ROI locations and then fuse them into a comprehensive feature representation.

**Figure 3:**
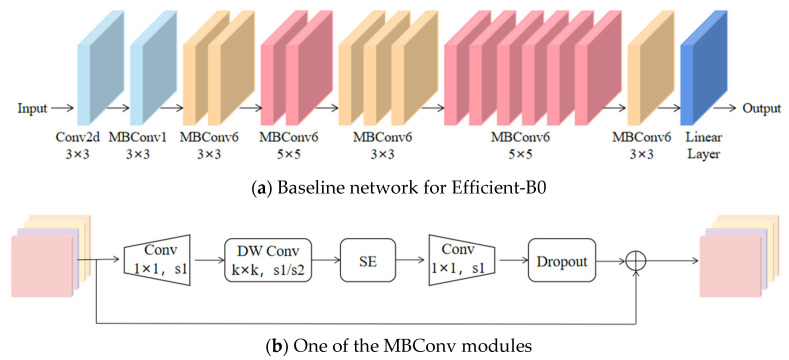
The framework structure diagram of EfficientNet. EfficientNet consists of a series of repetitive MBConv modules.

We employ the pre-trained EfficientNet [29] model for extracting features from ROIs. As shown in Figure 3, EfficientNet is an efficient neural network architecture, significantly reducing the parameter count and computational complexity while maintaining model accuracy. EfficientNet consists of a series of repetitive MBConv modules, where each MBConv uniquely integrates the SE module. Its final layer combines global pooling and a fully connected layer. EfficientNet encompasses various network structures, each contingent on different parameters, ranging from B0 to B7. For our classification model, we opted for EfficientNet-B0. Initially, we utilize a sequence of MBConv modules to extract features from ROIs. Subsequently, following global pooling, each face image yields a 1280 × N feature vector, where N represents the number of ROI features. Furthermore, we experimented with replacing the SE module with alternative attention mechanisms, but the enhancement was relatively modest.

We utilize the PyTorch 2.3.1 framework to pre-train and fine-tune the facial feature extraction process. We set the batch size to 16, ensuring that the final extracted feature vector has a shape of (16, 1280, N), where N represents the number of ROI features. For training our network, we employ the Stochastic Gradient Descent (SGD) optimization algorithm. We initialize the learning rate at 0.01, set the momentum to 0.9, and apply a weight decay of 0.0001. The entire training process spans a total of 50 epochs.

(c) The extracted feature vector ultimately assumes a shape of (16, 1280, N), where N can be either 6 or 5. Our analysis encompassed overall six ROI parts, two of which—the philtrum and chin—share similar color characteristics. We conducted ablation experiments to determine the optimal combination for classification. Following the flatten operation, the feature vector’s shape transforms to (16, 1280 × N), enabling its input into the fully connected layer for classification.

It is worth noting that we employed CNN to independently extract features from each ROI site. However, during the prediction phase, the initial input to the network remains a complete face image. Following ROI segmentation, CNN proceeds to extract and evaluate the respective features from each segmented region.

#### 2.2.2. Multi-Feature Splicing Classification Algorithm

The multi-feature splicing classification algorithm adopts the identical set of ROI features as its predecessor but with a distinct twist. In this iteration, we interlace images of distinct ROI segments and reshape them to create a novel data image.

**Figure 4:**
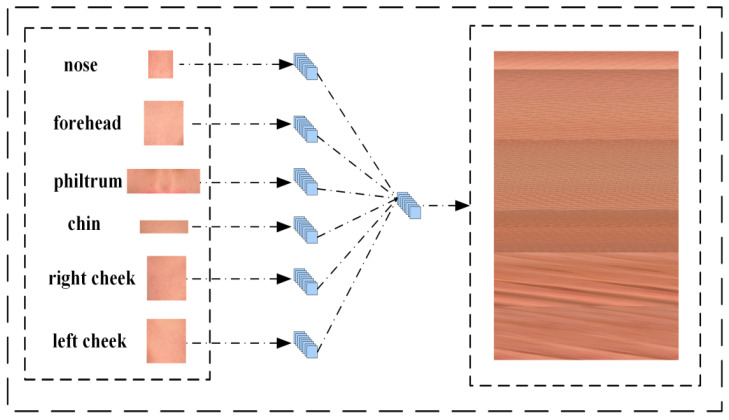
The framework of the multi-feature splicing classification algorithm.

Initially, we acquired segmented visuals of six ROI locations. To enhance the model’s training, we adjusted all the images with the same height-to-width ratio but different sizes to the same size. We opted for the median size as the standardized dataset measure, ensuring that color details remain intact despite the dimensional alteration. Six ROI images with different sizes were obtained after data processing. As shown in Figure 4, these images were first spread in the channel dimension, resulting in data with a shape of (h × w, 3). Then, the six ROI parts data of each face were merged in the first dimension. Finally, we obtain N sets of data with shape (6 × h × w, 3), where N is the number of faces. Of course, here the h and w of the six groups of ROI data are not the same.

To standardize the data, we transformed its dimensions to achieve a shape of (h, w, 3), where h and w are determined by the images of the six sets of ROI sites. This final data was then saved in image format, creating a novel dataset as illustrated in Figure 3. Additionally, we conducted ablation tests to identify which combinations of ROI features yielded the most accurate predictions.

#### 2.2.3. Multi-Feature Classification Algorithms with Machine Learning

**Figure 5:**
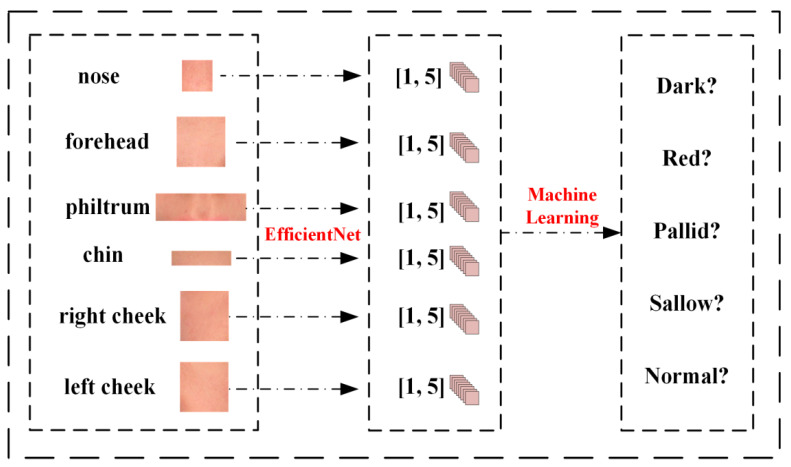
The framework of the multi-feature classification algorithms with machine learning.

As illustrated in Figure 5, the algorithm relies primarily on images of the ROI features. We leverage EfficientNet to train each of the six ROI datasets, ultimately deriving prediction weights for the respective sites. The trained model is then employed to predict ROI images, yielding six sets of predicted data. The predicted categories are compared against the actual categories, resulting in a numerical dataset.

Subsequently, we normalize and standardize the five categories, numbering them (dark, red, pallid, sallow, and normal) from 1 to 5, and segregating them into training and testing sets at a ratio of 8:2. A variety of machine learning techniques, encompassing K-Nearest Neighbors (KNN), Decision Tree, Random Forest, Linear Regression, Support Vector Machine (SVM), and XGBoost [30], are employed to train and predict the new dataset. Furthermore, ablation experiments are conducted to assess the optimal combinations of features and algorithms, aiming to enhance the overall predictive performance.

### 2.3. Evaluation Indicators

The model’s performance is evaluated using Precision (P), Recall (R), and F1-score. F1-score is the harmonic mean of precision and recall. The formula for P, R, and F1-score is as follows:

Here, TP (true positives) denotes correctly classified positive samples, FP (false positives) indicates misclassified positive samples, and FN (false negatives) refers to misclassified negative samples. Precision quantifies the probability that a model’s predicted positive classes are truly positive, whereas recall evaluates the model’s capability to identify all positive instances.

(1) $$P=\frac{TP}{TP+FP}$$

(2) $$R=\frac{TP}{TP+FN}$$

(3) $$F1-Score=2\times \frac{P\times R}{P+R}$$

## 3. Experimental Results

We trained and predicted the three algorithms and performed ablation experiments with different combinations of features. In addition, we also conducted correlation analysis and importance analysis for five ROI regions.

### 3.1. Results of Multi-Feature Fusion Classification Algorithm

First, we evaluated the feature extraction capabilities of different convolutional neural networks. We trained the models using a dataset comprising full facial images, and the results are presented in Table 1. The model with the highest accuracy was ResNet, and EfficientNet achieved slightly lower accuracy. However, EfficientNet has fewer parameters, enabling faster feature extraction. Moreover, EfficientNet boasts more novel architecture. Therefore, we opted for EfficientNet as our feature extraction network.

**Figure 6:**
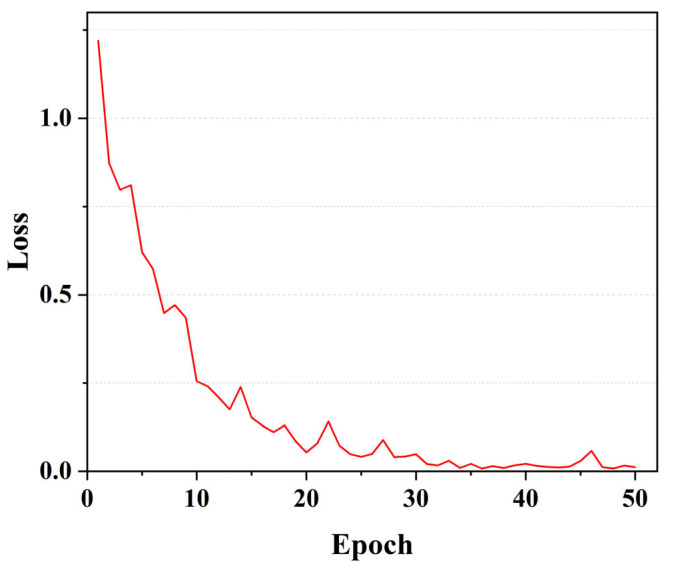
Loss function of the training process.

We initially assessed the efficacy of the multi-feature fusion classification algorithm. The results of this evaluation for the five specific categories under consideration are meticulously presented in Table 2. Notably, the precision for the sallow complexion category achieved a peak of 97.62%, while the recall for the red complexion category stood at 97.02%. Moreover, the F1-score for the pallid complexion category excelled, reaching 96.88%. These findings demonstrate the remarkable classification performance of our proposed facial complexion classification algorithm. The loss function during the training process is illustrated in Figure 6. The loss value drops rapidly with training and then gradually converges to a stable state, without any occurrence of overfitting.

### 3.2. Comparison of Different Algorithms and Ablation Experiments

To further assess the classification performance of our algorithm, we conducted comparative experiments with two facial complexion classification algorithms under identical conditions. Experiments 2-1, 2-2, and 2-3 represented ablation experiments of the second algorithm, employing three distinct feature combinations for training, similar to the first algorithm. A selection of these comparative experiments is outlined in Table 3. Experiment 1-1 involved eliminating grayscale features and solely relying on the three RGB color channels for feature fusion. Experiment 1-2 entailed directly concatenating the extracted features from the four channels, aligning with the pixel distribution. Experiments 1-3 and 1-4 served as ablation studies of our proposed algorithm, utilizing features from five distinct facial regions (nose, forehead, chin, right cheek, and left cheek) and all six regions for fusion, respectively.

As depicted in Table 4, all three experimental results of the multi-feature splicing classification algorithm achieved more than 90% accuracy. While the algorithm exhibited commendable classification results in experiments 2-1, 2-2, and 2-3, it still paled compared with the first algorithm. Feature fusion using three color channels alone yields slightly inferior classification results compared to incorporating grayscale channels. Specifically, in experiments 1-2, directly concatenating features based on pixel distribution resulted in the lowest accuracy of just 40.50%. The accuracies of 92.79% and 94.01% for experiments 1-3 and 1-4 show that the combination of nose, forehead, philtrum, right cheek, and left cheek features leads to the best classification results. Additionally, these experiments revealed the optimal combination of ROI features for classification.

**Figure 7:**
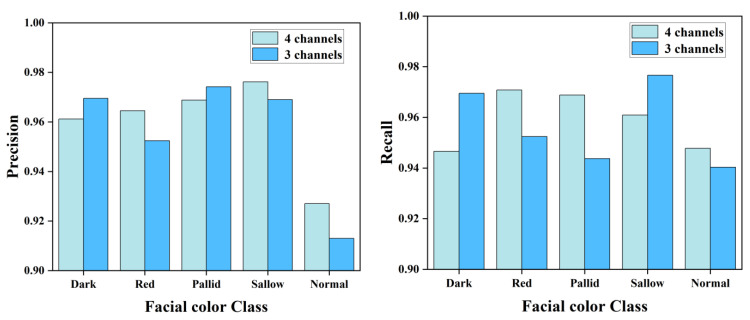
Visual presentation of results from 3- and 4-channel multi-feature fusion classification algorithms.

Furthermore, we presented the results of experiment 1-1 and 1-5 in the form of distinct bar charts to provide a more intuitive illustration of the classification effect. As depicted in Figure 7, the comparison reveals that the three-channel multi-feature fusion algorithm boasts a superior recognition accuracy for the dark category, whereas the four-channel approach excels in recognizing the red and normal categories. Notably, there is no significant disparity in the overall recognition accuracy between the two algorithms.

A separate experiment was performed for the third algorithm, starting with the use of the ROI dataset’s trained weights for prediction. Subsequently, the five categories (dark, red, pallid, sallow, and normal) were assigned numerical values ranging from 1 to 5. The predictive metrics pertaining to the six ROI features are presented in Table 5, indicating that the forehead achieved the highest combined accuracy of 95.15%, while the left cheek recorded the lowest accuracy of 81%.

**Figure 8:**
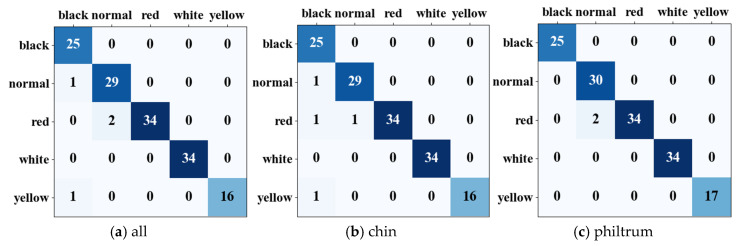
Confusion matrices for the classification results of the XGBoost algorithm.

We obtained a categorized dataset of prediction data, with values ranging from 1 to 5. Subsequently, we carried out the process of normalizing and standardizing this data to ensure uniformity and comparability across the dataset. We used various machine learning methods with a ten-fold cross-validation method for comparative training. The outcomes of the ablation experiments are detailed in Table 6, with the ‘chin’ column indicating the inclusion of chin in the five-feature set, and the ‘philtrum’ column following suit. Notably, the XGBoost algorithm emerged as the most accurate, achieving a 97.78% accuracy, where its composition of ROI features encompassed the philtrum. This remarkable result surpasses the performance of the first algorithm. As shown in Figure 8, one of the relatively superior classification results of the XGBoost algorithm is visualized using confusion matrices, which shows that the multi-feature classification of the philtrum region yields better results. Other machine learning algorithms that we tested yielded varying degrees of accuracy, with rates of 92.65%, 96.08%, 95.52%, 92.52%, 92.65%, and 94.19%, respectively.

After conducting an exhaustive comparison between the three classification algorithms, it was determined that the multi-feature classification combined with machine learning delivered the highest classification accuracy. Specifically, when integrated with the XGBoost algorithm, an impressive accuracy of 97.78% was attained. In contrast, the peak accuracy rates for the other two algorithms stood at 95.98% and 93.76%, respectively. This outcome can be attributed to the preliminary classification of facial features using the EfficientNet model, which preceded the application of machine learning techniques. Furthermore, XGBoost stands out as a potent and adaptable machine learning algorithm, renowned for its efficiency and exceptional performance. Additionally, we attempted to train the entire face image directly using the EfficientNet model, yielding a classification accuracy of 89.37%. However, this result pales in comparison to the efficacy of our proposed algorithm.

To validate the robustness and stability of the experimental results, we conducted multiple verifications using different data-splitting ratios and a range of experimental iterations. As shown in Table 7, we split the dataset according to ratios of 6:4, 7:3, and 8:2, respectively, and performed both 5-fold and 10-fold cross-validations. We incorporated the standard deviation metric to assess the stability of the experimental outcomes. The final results indicate that splitting the dataset in an 8:2 ratio along with conducting a five-fold cross-validation experiment yields the highest accuracy. However, to ensure the reliability of the results, we still adopted the outcomes of the 10-fold cross-validation experiment as the final accuracy rate. It is worth noting that the standard deviations for all experiments were low, indicating excellent stability in the experimental results.

We conducted a paired *t*-test to compare and analyze the performance of the multi-feature fusion and splicing algorithm with that of the multi-feature classification combined with machine learning. The performance difference between the two methods was found to be highly statistically significant (t = 2.851, *p* = 0.036). Additionally, we further calculated the effect size using Cohen’s d, which yielded a result of 1.829. This value far exceeds the conventional threshold for a large effect size (d > 0.8), indicating that the multi-feature classification combined with the machine learning algorithm not only significantly outperforms the other two algorithms but also holds substantial practical value. This result validates the effectiveness of our algorithm in facial complexion classification.

Finally, as shown in Table 8, we conducted a survey of some literature on facial complexion classification and found that the claimed accuracy rates were all lower than that of our proposed model. However, we were unable to obtain their source code and configuration environments, making it impossible to conduct further quantitative comparisons. Therefore, we have only conducted a comparison based on the accuracy metric, which clearly demonstrates the superiority of our model.

### 3.3. Correlation and Importance Analysis

**Figure 9:**
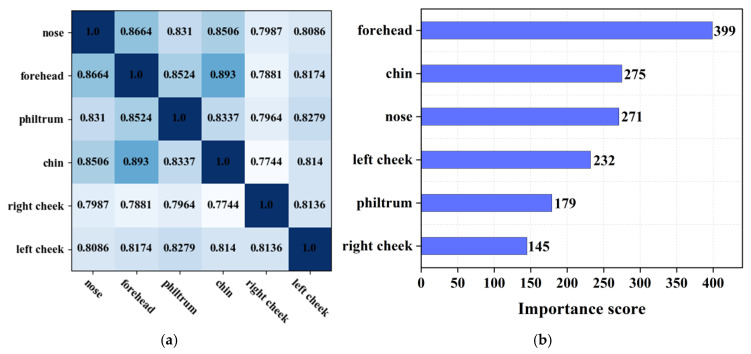
(**a**) Correlation analysis of 5 ROI features. (**b**) Importance analysis of 5 ROI features.

Moreover, we performed a comprehensive correlation and importance analysis encompassing six ROI regions. Initially, we employed the Pearson correlation coefficient to quantify the degree of association between two distinct ROI regions. This coefficient ranges from 0 to 1, where a value closer to 1 signifies a stronger correlation. Figure 9a presents the correlation heatmap, wherein the intensity of color depicts the strength of the correlation; darker shades indicate a higher correlation, while lighter shades represent a weaker correlation. For example, from this visualization, it is evident that the chin exhibits a significant correlation with the forehead and a weaker correlation with the right cheek.

To assess the importance of each ROI region, we leveraged the XGBoost algorithm to quantify their contribution to the overall forecasting accuracy. As depicted in Figure 9b, the forehead emerged as a highly significant region, whereas the philtrum and right cheek contributed relatively less. Additionally, we observed a distance in the importance score attributed to the left and right cheeks, which necessitated a more thorough investigation of the initially collected dataset. Our examination unveiled that certain patients had a preference for being photographed with their faces slightly turned, leading to an asymmetry in the left and right facial structures within the gathered data. Notably, this asymmetry did not exert influence on the face complexion classification outcomes.

## 4. Conclusions

The human face, akin to a mirror, provides a window into the body’s underlying diseases and lesions. To address the challenge of accurately categorizing facial complexion, we introduce three innovative multi-feature classification algorithms based on convolutional neural networks. These algorithms are trained and make predictions by learning from different facial ROI features. Among the proposed methods, the multi-feature fusion algorithm excelled, achieving a commendable classification accuracy of 95.98%. The multi-feature splicing algorithm attained a maximum accuracy of 93.76%. Notably, the algorithm that combines multi-feature CNN with the XGBoost machine learning algorithm surpassed all others, boasting an impressive accuracy of 97.78%. Furthermore, we conducted correlation and importance analysis to gain deeper insights into the impact of various ROI features on the classification outcomes. In summary, the multi-feature classification algorithms verify the effectiveness of multidomain combinations and improve the accuracy of facial complexion classification. The bio-inspired approach lays an important foundation for the development of non-invasive diagnostic and health monitoring technology.

## Figures and Tables

**Table 1:** Comparison of feature extraction capabilities of different convolutional neural networks.

Model	Accuracy	Precision (Average)	Recall (Average)	F1-Score (Average)	Params	Duration (One Epoch)
EfficientNet	89.37%	90.34%	88.62%	89.53%	5.3 M	52 s
VGGNet	53.53%	53.12%	54.73%	52.77%	138 M	72 s
MobileNet	51.41%	56.87%	48.96%	46.47%	3.4 M	47 s
ResNet	90.14%	90.91%	90.53%	90.33%	25.5 M	61 s
DenseNet	86.62%	90.61%	87.21%	86.87%	8 M	55 s
ConvNeXt	88.03%	89.17%	87.91%	88.15%	28 M	68 s

**Table 2:** Evaluation results of multi-feature fusion classification algorithm.

Indicators	Dark	Red	Pallid	Sallow	Normal
Precision	96.12%	96.45%	96.88%	97.62%	92.70%
Recall	94.66%	97.02%	96.88%	96.09%	94.78%
F1-score	95.38%	96.73%	96.88%	96.85%	93.73%

**Table 3:** Comparison experiments numbered from 1-1 to 2-3.

Algorithm	Comparison	Experiment
Multi-feature fusion classification algorithm	1-1	3 channels (RGB)
	1-2	pixel splicing
	1-3	with chin parts
	1-4	all 6 parts
	1-5	4 channels (RGB + Gray)
Multi-feature splicing classification algorithm	2-1	with philtrum parts
	2-2	with chin parts
	2-3	all 6 parts

**Table 4:** Evaluation results of comparison experiments.

Comparison	Accuracy	Precision (Average)	Recall (Average)	F1-Score (Average)
1-1	95.56%	95.52%	95.65%	95.59%
1-2	40.5%	42.01%	39.54%	36.15%
1-3	92.79%	92.79%	92.91%	92.80%
1-4	94.04%	94.43%	93.84%	94.07%
1-5	95.98%	95.95%	95.88%	95.91%
2-1	93.76%	93.67%	93.96%	93.73%
2-2	90.01%	90.62%	89.91%	89.90%
2-3	90.29%	90.64%	90.18%	90.28%

**Table 5:** Prediction results for six ROI features.

ROI	Accuracy	Precision (Average)	Recall (Average)	F1-Score (Average)
nose	92.23%	92.29%	92.15%	92.18%
forehead	95.15%	95.43%	95.03%	95.19%
philtrum	91.12%	90.99%	91.05%	90.96%
chin	90.98%	90.73%	90.88%	90.79%
right cheek	90.29%	90.23%	90.13%	90.13%
left cheek	81.00%	81.51%	81.23%	80.82%

**Table 6:** Comprehensive accuracy of different machine learning algorithms.

Model	All	Chin	Philtrum
KNN	92.81%	93.97%	92.65%
Decision tree	95.58%	94.91%	96.08%
Random forest	95.52%	95.24%	95.52%
Linear1	92.33%	92.28%	92.57%
Linear2	92.67%	92.41%	92.65%
SVM	94.53%	94.19%	94.19%
XGBoost	97.23%	96.13%	97.78%

**Table 7:** Results of different dataset division ratios and number of experiments.

Ratio	6:4		7:3		8:2	
Fold	5	10	5	10	5	10
Accuracy	97.57	97.19	97.96	97.51	98.23	97.78
Standard deviation	0.43	0.79	1.03	0.99	0.88	1.11

**Table 8:** The comparison of our model with those in other papers.

Model	Accuracy
Ours	97.78%
Zhao et al. [7]	86.89%
Lin et al. [10]	91.03%
Hou et al. [21]	93%
Lin et al. [23]	83.96%
Zhao et al. [24]	94.12%

## Data Availability

Data will be made available on request.
